# Introducing GWAStic: a user-friendly, cross-platform solution for genome-wide association studies and genomic prediction

**DOI:** 10.1093/bioadv/vbae177

**Published:** 2024-11-12

**Authors:** Stefanie Lück, Uwe Scholz, Dimitar Douchkov

**Affiliations:** Department of Breeding Research, Leibniz Institute of Plant Genetics and Crop Plant Research (IPK), OT Gatersleben, D-06466 Seeland, Germany; Department of Breeding Research, Leibniz Institute of Plant Genetics and Crop Plant Research (IPK), OT Gatersleben, D-06466 Seeland, Germany; Department of Breeding Research, Leibniz Institute of Plant Genetics and Crop Plant Research (IPK), OT Gatersleben, D-06466 Seeland, Germany

## Abstract

**Motivation:**

Advances in genomics have created an insistent need for accessible tools that simplify complex genetic data analysis, enabling researchers across fields to harness the power of genome-wide association studies and genomic prediction. GWAStic was developed to bridge this gap, providing an intuitive platform that combines artificial intelligence with traditional statistical methods, making sophisticated genomic analysis accessible without requiring deep expertise in statistical software.

**Results:**

We present GWAStic, an intuitive, cross-platform desktop application designed to streamline genome-wide association studies and genomic prediction for biological and medical researchers. With a user-friendly graphical interface, GWAStic integrates machine learning and traditional statistical approaches to support genetic analysis. The application accepts inputs from standard text-based Variant Call Formats and PLINK binary files, generating clear graphical outputs, including Manhattan plots, quantile–quantile plots, and genomic prediction correlation plots to enhance data visualization and analysis.

**Availability and implementation:**

Project page: https://github.com/snowformatics/gwastic_desktop; GWAStic documentation: https://snowformatics.gitbook.io/product-docs; PyPI: https://pypi.org/project/gwastic-desktop/

## 1 Introduction

The field of genomics has rapidly advanced, creating a need for tools that simplify genetic data and utilize emerging technologies. Genome-wide association studies (GWAS) (Uffelmann et al. 2021) have transformed our understanding of complex traits by associating genetic markers with diseases like heart disease, diabetes, and cancer, leading the way to targeted therapies and personalized medicine (Tam et al. 2019). In agriculture, GWAS aids in identifying traits for drought and pest resistance in crops, facilitating the development of resilient and high-yield varieties crucial for food security and sustainable agriculture. Additionally, these studies help transfer biodiversity insights into agricultural practices, promoting genetic diversity and enhancing ecosystem resilience.

Genomic prediction (GP) has emerged as a key tool in genetics, allowing for the prediction of traits based on genetic information, greatly benefiting both plant breeding and medical research (Hickey et al. 2017). In agriculture, it streamlines the selection process for desirable traits, speeding up the breeding of superior crop varieties. In medicine, GP offers insights into genetic predispositions to diseases, enabling early interventions and customized treatments.

Despite their promise, the complexity and volume of GWAS and GP data require sophisticated yet challenging-to-use software tools. This article introduces GWAStic, a versatile, cross-platform application tailored for GWAS and GP analysis. Designed to be user-friendly, GWAStic bridges the gap between artificial intelligence (AI) and traditional statistical methods for genetic analysis. By focusing on ease of use, GWAStic makes the advanced fields of GP and GWAS accessible to researchers without the need for deep expertise in complex statistical software. Developed in Python and available through the Python Package Index (PyPI), it supports use across Microsoft Windows, macOS, and Linux, enhancing collaborative research and expanding access to advanced GP tools. While GWAStic offers substantial analysis capabilities, it is not intended to replace comprehensive statistics packages but rather to provide researchers easy access to genomic analysis tools, integrating genomic insights into their work with less of a learning curve.

## 2 Software development and availability

GWAStic is developed with the popular Python language (v.3.9), known for its versatility and robust scientific computing libraries. To ensure ease of access and installation, GWAStic is available via PyPI (Lueck 2024a), a well-established platform for managing Python packages. This inclusion further simplifies the process for users to set up and start utilizing GWAStic on their preferred operating system, Windows, macOS, or Unix. GWAStic’s codebase and documentation are hosted on GitHub (Lueck 2024b). The minimum disk space required for an Anaconda installation with the gwastic software package is ∼8 GB, while a pure Python installation with the gwastic software package requires ∼2 GB.

## 3 Features

### 3.1 Algorithms

**Figure 1.**
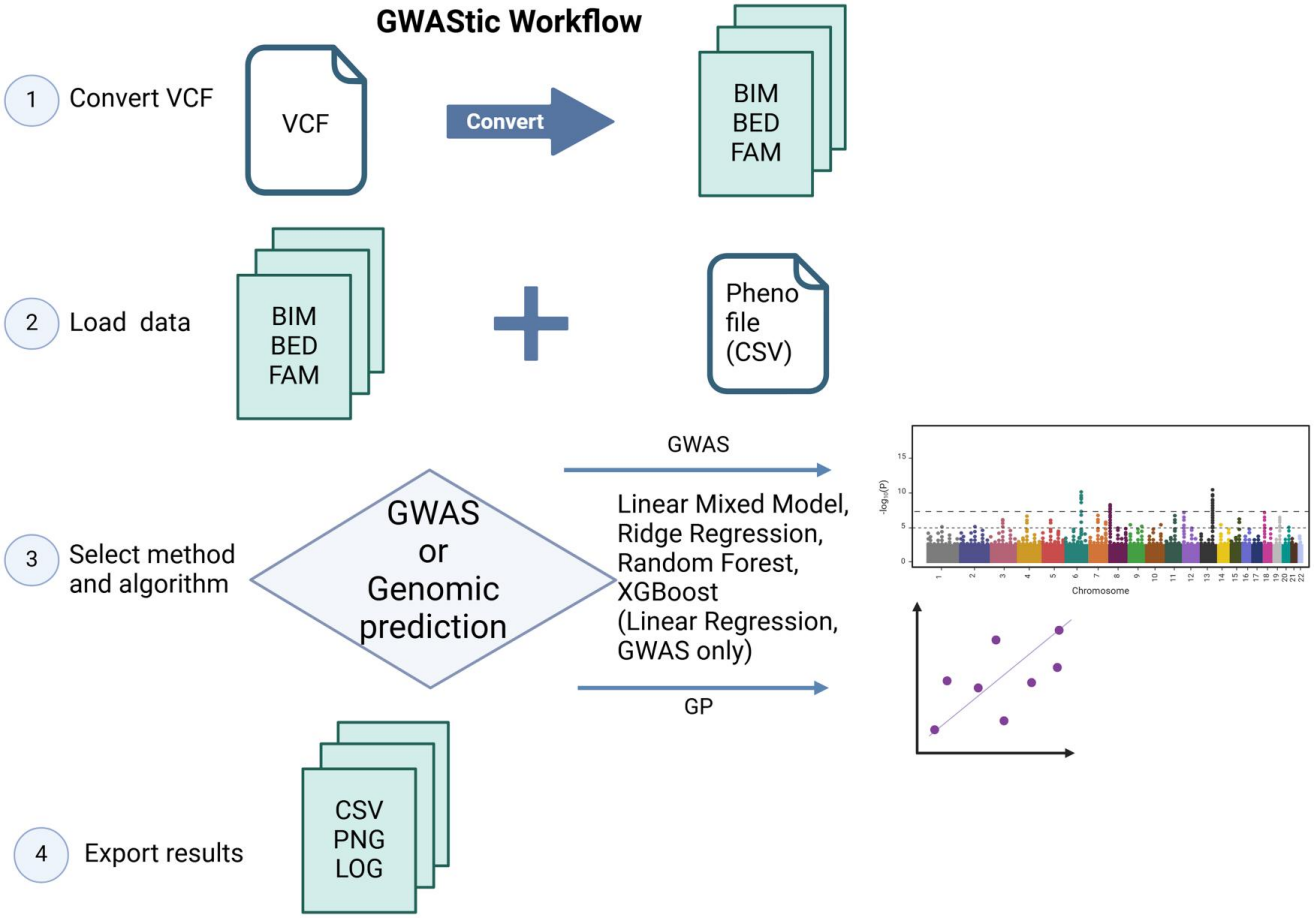
GWAStic workflow. Step 1: Converting the VCF files to BIM, BED, and FAM files. If available, the BIM, BED, and FAM files can be imported directly. Step 2: Load the phenotypes data file (in CSV format) and the genotypes binary BED file. Ensure that the corresponding BIM and FAM files are in the same folder as the BED file. Step 3: Choose the method: GWAS or Genomic prediction), and select the algorithm: Linear Mixed Model, Random Forest, XGBoost, Ridge Regressions, and Linear Regression (available only for GWAS). Perform the analysis. Step 4: Export the tabular and graphical results (optional).

GWAStic features various methods for conducting GWAS and GP, offering researchers a versatile toolkit for cutting-edge genetic analysis (Fig. 1).

The linear regression (LR) method uses the FaST- linear mixed model (LMM) package for Python (Lippert et al. 2011). It provides a straightforward way to identify potential associations between genetic variants and traits without accounting for genetic relatedness. To overcome this method’s limitations, especially its inability to account for population structure, GWAStic employs LMMs with automatically generated kinship matrix using the same FaSt-LMM package, significantly improving the precision of association detection by adjusting for genetic background noise.

Expanding beyond conventional statistical models, GWAStic incorporates machine learning (ML) with the inclusion of Random Forest (RF) (Breiman 2001) and Ridge Regression (RR) (Hilt and Seegrist 1977) via the Scikit-learn library (Pedregosa et al. 2011) and XGBoost (XGB) (Chen and Guestrin 2016) through the XGB Python Package (Cho 2024). RF succeeds in dissecting complex, non-linear relationships among genetic variants, offering a robust approach for GWAS (Stephan et al. 2015). XGB complements this by applying sophisticated regularization techniques to control overfitting, thereby enhancing the reliability and accuracy of GWAS outcomes. RR is a ML technique for LR that also adds a regularization term to the loss function to help prevent overfitting. Collectively, these five methodologies assemble a versatile toolkit, enabling researchers to explore the genetic foundations of traits and diseases with unparalleled precision and comprehensiveness. More details about the different algorithms and their recommended application is provided in the online documentation (link in the Abstract).

### 3.2 Customizable settings for personalized analysis

GWAStic offers customizable parameters for diverse research needs, including modifying significance thresholds and data conversion settings. Key features include adjusting minor allele frequency (MAF) for allele occurrence analysis, managing missing genotype rate for incomplete genetic data, and setting training size for ML-based algorithms.

The GWAStic also includes useful utilities to facilitate the workflow and visualize the results.

### 3.3 Variant Call Format to BED/BIM/FAM file converter

GWAStic integrates a converter for transforming Variant Call Format (VCF) files into PLINK (Purcell et al. 2007) BED/BIM/FAM files, where the BED file contains matrix genotypes in binary format, BIM lists information about the variants, and FAM lists information about the samples. This conversion capability is critical, as VCF is a standard format for storing gene sequence variations, while BED/BIM/FAM files are widely used in various genetic analysis software. At this point, the data can be filtered for MAF and missing genotype rate. The VCF to BIM file converter in GWAStic simplifies the workflow for researchers, enabling them to use data from a wide range of sources and platforms without needing external conversion tools.

### 3.4 Visualization tools

The software includes tools for generating Manhattan and QQ plots, two of the most common visual representations in GWAS. Manhattan plots provide a clear visualization of genetic variant significance across the genome, while QQ plots assess the distribution of *P*-values, identifying deviations that may indicate issues such as population stratification or cryptic relatedness. GWAStic also offers options to generate comprehensive summary and statistical plots for both genotypic and phenotypic data. For genotypic data, the software provides histograms of MAF, marker density, and heterozygosity, along with principal component analysis (PCA) plots, heritability plots, and hierarchically clustered kinship matrix plots. Phenotypic data are visualized through various histograms and plots that illustrate the frequency and distribution of observed phenotypes. These intuitive tools are essential for identifying and interpreting key genetic associations. For GP, the software provides a Bland–Altman Plot as a tool for evaluating model performance alongside a correlation plot with predicted and actual phenotypic values with Pearson correlation, facilitating a comprehensive interpretation of the results.

## 4 Validation

In a practical demonstration of GWAStic’s capabilities, we conducted an extensive analysis using a comprehensive barley dataset showcasing the effectiveness and versatility of the different GWAS methodologies embedded in our software. Each method was tested for accuracy, computational efficiency, and ability to handle the complexity of the barley genome.

We have used a subset of data from a recent study (Milner et al. 2019) focusing on the genetic basis of barley traits. The genotypic data were filtered by applying a genotyping rate cutoff of 0.02 and a MAF threshold of 0.05. This resulted in a curated dataset comprising 949 174 single nucleotide polymorphisms (SNPs). A random subset of 147 accessions from the Core 200 collection in the same study with available row-type phenotype data was selected. This phenotype describes the arrangement of kernels on the spike of the barley plant, specifically distinguishing between two-rowed and six-rowed barley—a crucial morphological and agricultural trait. In this GWAS study, the five distinct methods—XGB, RF, RR, LR, and LMM—were employed to validate the peaks of two row-type associated barley genes previously identified in (Milner et al. 2019) (Supplementary Fig. S1, Supplementary Tables S1–S5).

LMM emerged as the slowest technique, while LR proved to be the fastest. However, it is noteworthy that LR exhibited the highest noise levels. Despite these variances in speed and noise, all five methods successfully confirmed the existence of the two gene peaks.

We strategically excluded 30 random samples from the 147 accessions to validate the GP methods. For each method, we then trained 10 genomic models using the remaining samples. This approach allowed us to predict the phenotypes of the excluded 30 samples, providing a robust assessment of each method’s predictive accuracy (Supplementary Tables, S10). The online documentation provides a detailed explanation of the training and validation datasets (link in the Abstract).

For the analysis involving LR and LMM, our study used the default configurations of the Fast-LMM library. The setups for RF and XGB were adjusted to a training-to-test ratio of 70/30. Moreover, each model was constructed with 200 trees and a maximum depth of 3, achieving an optimal equilibrium between model intricacy and mitigating the risk of overfitting. The other parameters for these models adhered to the default values specified in the Python Scikit-Learn library. The experimental process was repeated 10 times with randomized 70/30 splits. Notably, after each iteration, we summed up the feature importance scores across all 10 ML models for each SNP, providing a cumulative insight into genetic markers’ relevance. This strategy was essential for achieving distinct peaks and minimizing noise, thereby enhancing the reliability of the findings. However, the mean and median figure importance are also available as scoring approaches for marker relevance in the ML methods.

To evaluate the performance of all methods across different datasets, alongside the barley dataset, we tested a smaller *Arabidopsis thaliana*-derived dataset, which includes 58 accessions and 385 649 SNPs (Vogt et al. 2022). Additionally, we tested two synthetically generated datasets, each containing 2000 samples and 90 010 markers, designed for quantitative and qualitative phenotypes (Lueck and Douchkov 2024). All applied methods reliably detected the designed marker associations in all datasets (Supplementary Figs S1–S3). The analysis was performed using Python 3.9 on a Windows 10 platform powered by an Intel Core i7-9700 processor with 32 GB RAM.

### 4.1 Performance test

The GWAStic tool is designed to make GWAS and GP analysis accessible for small to medium-sized datasets (up to a few thousand individuals and several million SNPs) on a desktop computer or laptop. To provide a performance estimate of different methods across various dataset sizes, we conducted performance tests, with the results detailed in Supplementary Table S12. While all implemented algorithms can perform these tasks on a desktop PC, there are notable differences in performance. For example, the LMM algorithm is more efficient with large sample sizes and smaller numbers of SNPs, whereas others, such as XGB, perform better with larger numbers of SNPs but fewer samples.

### 4.2 Comparison to previously published tools

Recent advances in genomics have led to the development of numerous genome analysis tools. However, the majority of these tools have a steep learning curve and are designed for advanced users. Conversely, there is a relative shortage of easy-to-use tools for the analysis of less complex data. Besides GWAStic, some recently developed tools aim to bridge this gap. To compare their capabilities and functionalities, we selected a recently published tool with similar aims: *vcf2gwas* (Vogt et al. 2022). The *vcf2gwas* tool is a Python API for the well-known GEMMA package (Zhou and Stephens 2012), offering a convenient command line interface for performing all steps of a traditional GWAS workflow. GWAStic and *vcf2gwas* share several features, including an easy interface, VCF conversion to BED/BIM/FAM format, quality filtering for marker data, multiple methods for GWAS, and graphical output (see Supplementary Table S11). However, there are also differences that may give one tool an advantage over the other, depending on the user’s needs and preferences.

One of the main differences is that GWAStic provides a graphical user interface (GUI), offering intuitive use, whereas *vcf2gwas* is a command-line tool with more options for workflow automation.

Another key difference is that GWAStic provides several methods for GP and ML algorithms, which are not available in the *vcf2gwas* tool.

In terms of performance, GWAStic outperforms *vcf2gwas* in run time for GWAS analysis using LMM models by ∼30%.

In conclusion, both tools have their overlaps and specific features, providing easy-to-use interfaces. Users who prefer a GUI may select GWAStic, while those who prefer the flexibility of a command line interface may choose *vcf2gwas*.

## 5 Results

In evaluating GWAStic with the barley dataset, we observed clear advantages and distinctions between the algorithms. The LR model emerged as the fastest and simplest, yet its lack of population structure correction led to increased noise in the results. Conversely, the LMM, incorporating kinship matrices, excelled in mitigating false positives by correcting for population structure and relatedness. RR adds a penalty to the magnitude of the coefficients in a linear model, helping to keep the model more stable and prevent overfitting. These linear methods may not suit complex, non-linear phenotypes influenced by multiple minor-effect loci.

ML approaches, such as RF and XGB, are believed to have strong potential in capturing complex non-linear relationships and gene interactions, which could offer valuable insights into trait heritability and genetic architecture (Elgart et al. 2022). Utilizing these five GWAS methodologies, our research confirmed two genomic regions previously associated with the phenotype, indicative of strong associations with barley row-type genes, VRS1 (HvHOR) and INT-C. VRS1 is crucial for determining barley’s row-type, playing a key role in barley morphology. The simultaneous detection of INT-C underscores the complex genetic dynamics in barley row-type development. In addition to the barley dataset, we tested synthetic datasets that included marker associations with both quantitative and qualitative traits. All the GWAS methods tested were able to reliably detect the designed associations. However, there were notable differences in performance across the different approaches applied to various datasets. Generally, the LMM method was significantly faster for datasets with many samples (2000) but a relatively lower number of markers (90 010), consistent with the original aim of the Fast-LMM library (Lippert et al. 2011). In contrast, the XGB method was twice as fast as LMM when using a dataset with fewer individuals (147) but a much larger number of markers (∼1 million).

However, the accuracy of AI-based methods heavily relies on parameter selection and multiple iterations with varied test–train splits, a critical approach given the dataset’s size. This necessitates careful data handling to achieve reliable insights.

GP models, capturing the cumulative effects of genetic variations, promise more accurate predictions for complex traits than GWAS. Nonetheless, complex non-linear phenotypes with epistatic interactions pose challenges to LR models like Genomic best linear unbiased prediction. XGB is considered particularly effective in analyzing complex non-linear and epistatic interactions, as it can handle high-dimensional data, apply gradient boosting for error correction, employ regularization to reduce overfitting, and potentially model gene interactions more effectively than some linear approaches. This proficiency makes XGB especially potent in GP and association studies, where trait genetics are typically complex and multi-layered.

## 6 Conclusion

In our evaluation of GWAStic on the barley dataset, we compared the performance of linear models (LR, LMMs, and RR) with ML approaches (RF and XGB). The LR model was fast, but its inability to account for population structure led to noisier results. The LMM improved upon this by adjusting for population structure, reducing false positives, and maintaining strong performance with large sample sizes and fewer markers, though it slowed considerably with large marker arrays.

ML models, particularly RF and XGB, showed potential for identifying complex, non-linear relationships and genetic interactions, which could provide deeper insights into trait genetics. These models were also notably faster than the LMM, specifically when dealing with smaller sample sizes supported by many markers—an increasingly important advantage given the advances in genomics and rising marker density.

Both approaches successfully identified all genomic regions significantly associated with the relevant phenotypes. However, the effectiveness of AI-based methods depended heavily on precise parameter selection and iterative testing, particularly with smaller datasets.

## Supplementary Material

vbae177_Supplementary_Data

## Data Availability

The data underlying this article are available in Zenodo at https://zenodo.org, and can be accessed with doi: 10.5281/zenodo.13695229.
