# Supplementary material for: Introducing GWAStic: a user-friendly, cross-platform solution for genome-wide association studies and genomic prediction
Source: Bioinform Adv. 2024 Nov 12;4(1):vbae177. doi: 10.1093/bioadv/vbae177 (PMC11643344; doi:10.1093/bioadv/vbae177)

Supplementary Figure S1: Marker association significance to the row type phenotype (barley dataset, 147 samples, 949,174 SNPs) using different methods.
For the SNP values please see the Supplementary Tables, S1-S5

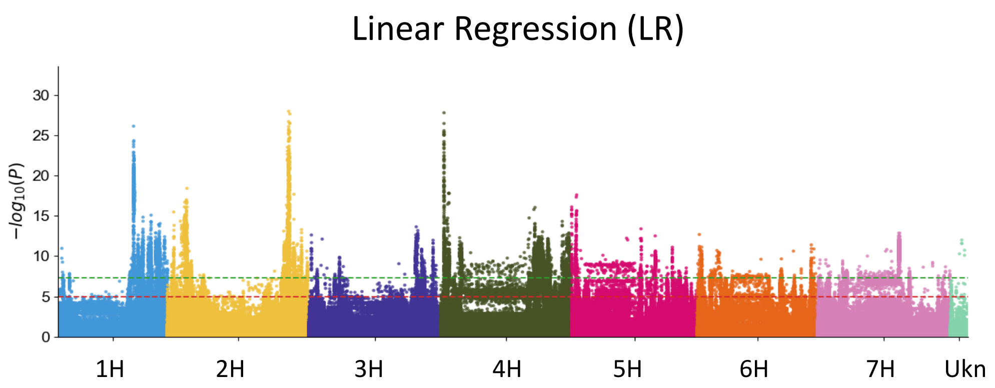

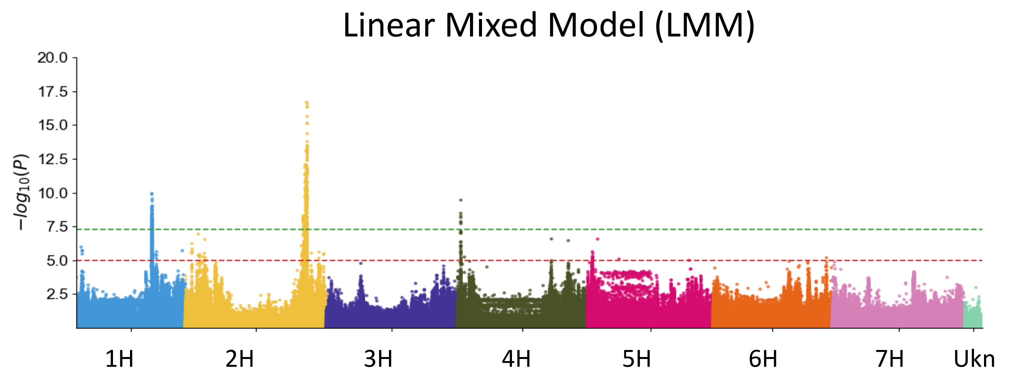

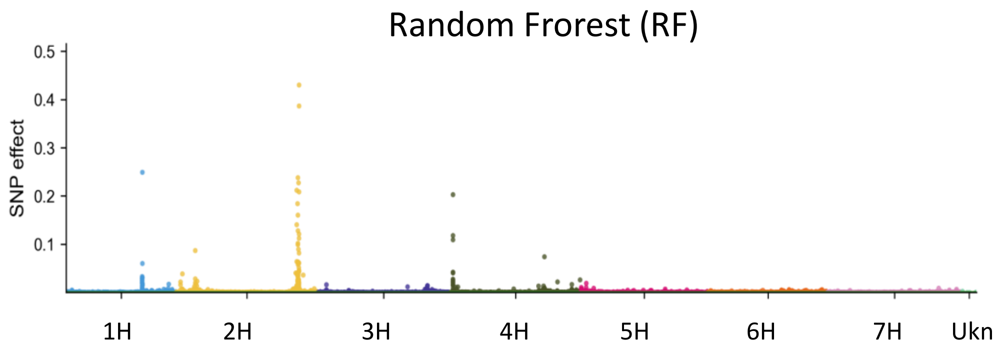

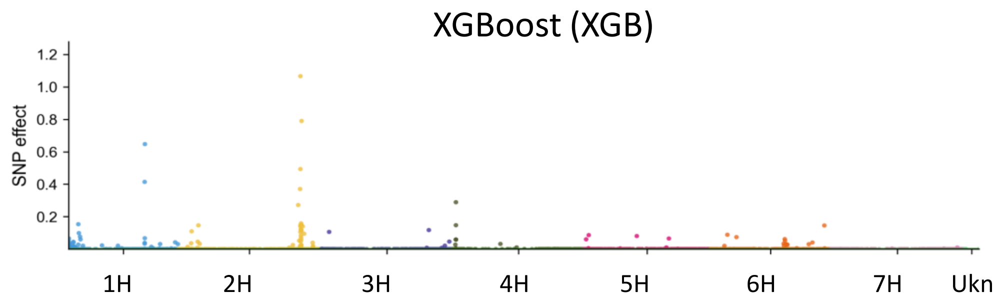

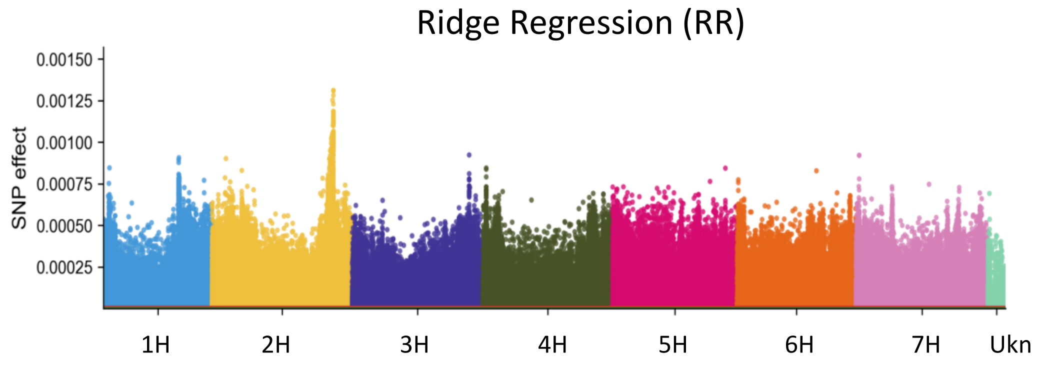

Supplementary Figure S2: Marker association significance in the synthetic dataset with qualitative (binary) phenotype.

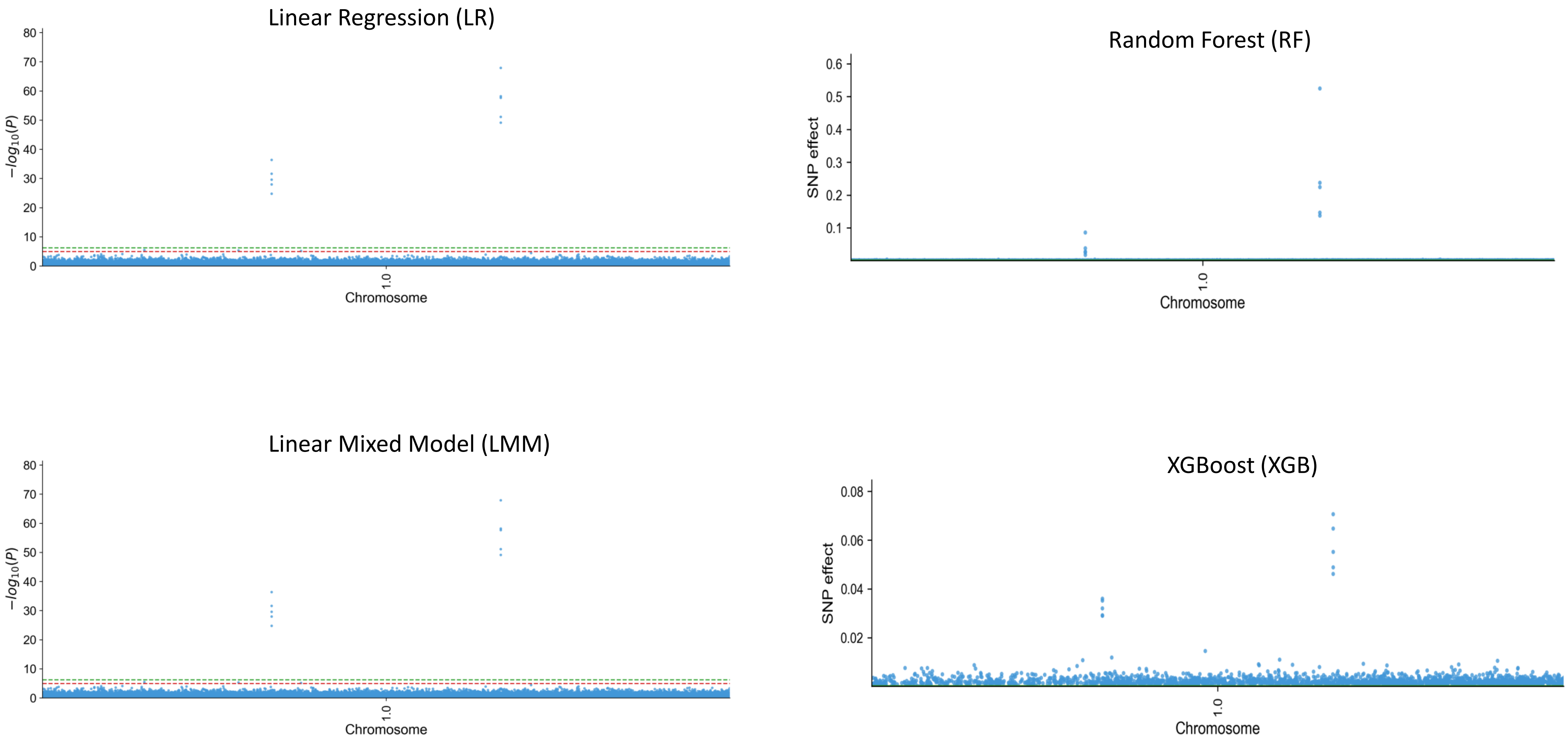

Supplementary Figure S3: Marker association significance in the synthetic dataset with a quantitative phenotype.

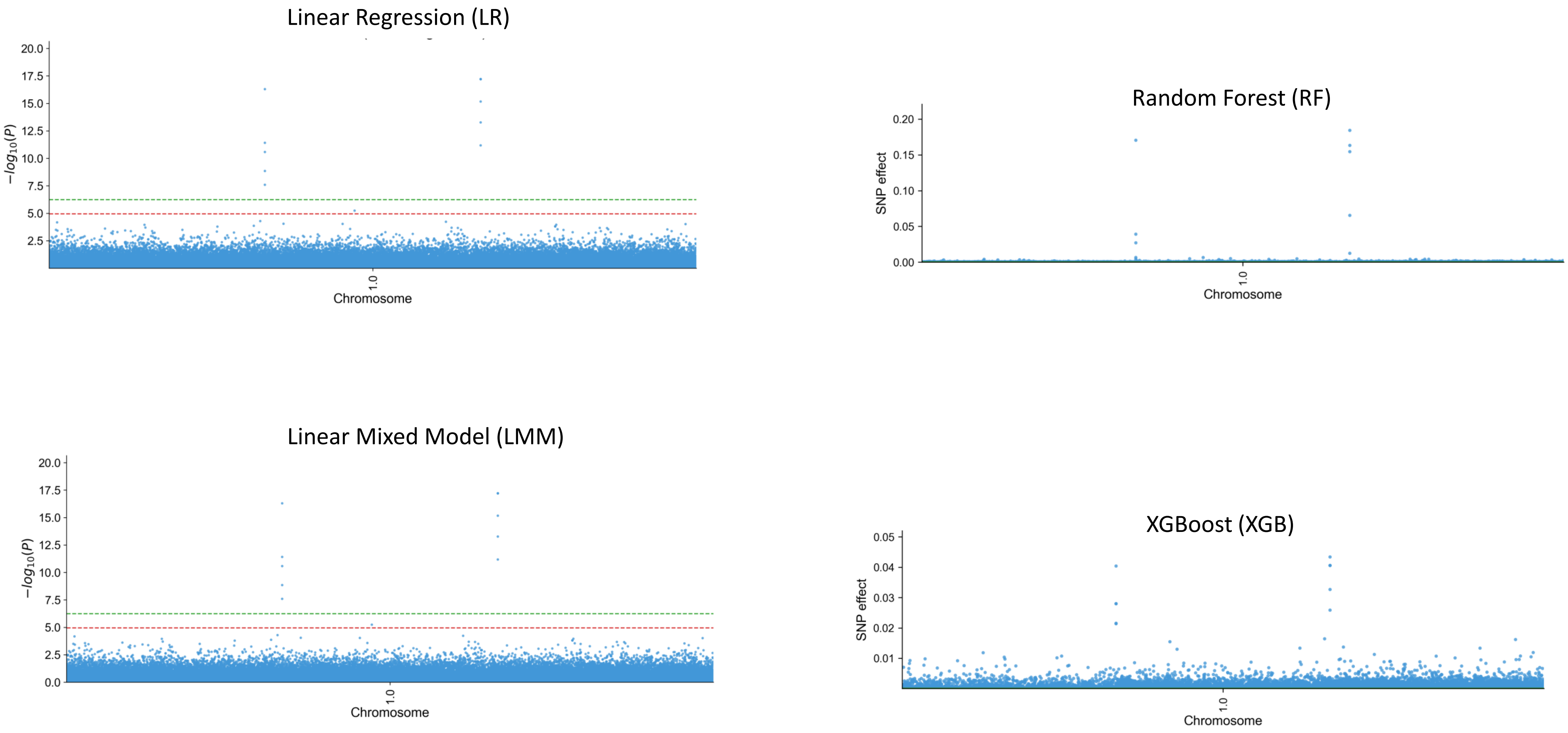

Supplement: vbae177_Supplementary_Data [file vbae177_supplementary_data.zip › Supplementary_Figures.pdf]
